# Attempt to visualize terminal structure on a specific facet in polymer–metal complex nanocrystals†

**DOI:** 10.1039/c8ra02165a

**Published:** 2018-05-03

**Authors:** Ryuju Suzuki, Tsunenobu Onodera, Hitoshi Kasai, Hidetoshi Oikawa

**Affiliations:** Research and Development Center for Marine Biosciences, Japan Agency for Marine-Earth Science and Technology (JAMSTEC) 2-15 Natsushima-cho Yokosuka 237-0061 Japan ryujus@jamstec.go.jp +81-46-867-9668; Institute of Multidisciplinary Research for Advanced Materials, Tohoku University Katahira 2-1-1, Aoba-ku Sendai 980-8577 Japan

## Abstract

We have successfully visualized the surface terminal structure of polymer–metal complex [{Cu_2_(μ-Br)_2_(PPh_3_)_2_}(μ-bpy)]_*n*_ nanocrystals (NCs) using Prussian blue (PB) nanoparticles (NPs). From TEM observation and analysis of the electron beam diffraction pattern, it was found that the (010) plane had grown well, and that the terminal ends of main chains would be located on the (010) plane of the present NCs as a dangling bond. Actually, PB NPs were selectively adsorbed on the (010) plane of [{Cu_2_(μ-Br)_2_(PPh_3_)_2_}(μ-bpy)]_*n*_ NCs. This fact clearly means bipyridine ligands having a nitrogen-terminal located on the surface of the (010) plane would coordinate and bind to Fe ions in PB NPs, which would lead to a new class of polymer–metal complex NCs materials.

## Abstract 2

We have successfully visualized the surface terminal structure of polymer–metal complex [{Cu_2_(μ-Br)_2_(PPh_3_)_2_}(μ-bpy)]_*n*_ nanocrystals (NCs) using Prussian blue (PB) nanoparticles (NPs).

## Graphical abstract

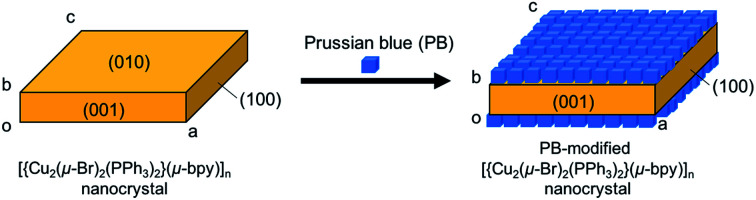

## 1. Introduction

Nanocrystals (NCs) have a large specific surface area, compared to the bulk crystal, and it is important to utilize the surface of the NCs so as to further develop and control the novel properties. In order to obtain the large benefit of the surface effect, we have to understand deeply the surface structure as well as physicochemical properties on the surface of NCs, and consider and design elaborately the surface interaction with molecules such as modifiers. It is, however, often difficult to not only control but also characterize the surface structure and properties of NCs. In general, since un-saturated bonds (or dangling bonds) are hardly exposed on the surface of organic bulk crystals and/or organic NCs, surface modification is typically performed through physical adsorption,^1–4^ instead of chemical adsorption induced by covalent bond formation.

So, we have strategically focused on the peculiar crystal structure of a polymer–metal complex (PMC).^5^ The main chains of PMC grow one-dimensionally in the crystal state. That is to say, the terminal end of the main chain is either a coordinatively-unsaturated center metal or ligand molecules in the present PMC and would be exposed on a specific surface, *i.e.*, specific facet, in the crystal state as a dangling bond. In a similar manner, terminal structure and end-groups often affect remarkably physicochemical properties in common polymer materials, and their assignment and quantitative analysis should be important in polymer science and engineering.^6–11^ In addition, PMC in a solid state shows luminescence from metal-to-ligand charge transfer (MLCT) excitation states, and the emission energy depends on energy level of π* orbital of ligand.^5^ In the previous study,^12^ we have fabricated various PMC NCs and changed the luminescence properties by the size effect. In PMC NCs, the energy level of π* orbital of bipyridine ligand was changed and affected the luminescent color of the NCs. Following these outcomes, we have attempted to reveal the surface properties and effects in PMC NCs at the present stage.

**Fig. 1:**
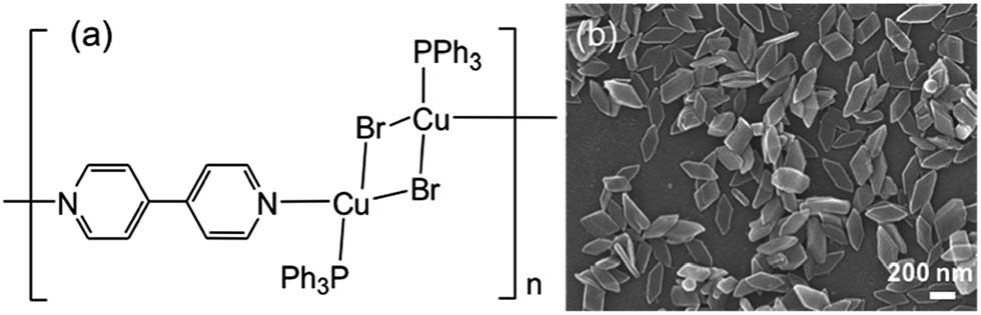
(a) Chemical structure of polymer–metal complex [{Cu_2_(μ-Br)_2_(PPh_3_)_2_}(μ-bpy)]_*n*_ (PMC as described in the text), and (b) SEM image of PMC NCs.

In the present article, we have chosen [{Cu_2_(μ-Br)_2_(PPh_3_)_2_}(μ-bpy)]_*n*_ NCs (Fig. 1),^12^ because of their well-defined parallelogram plates, and attempted to visualize terminal structure on specific facet in the present PMC NCs using a suitable modifier. If coordinatively-unsaturated bipyridine unit is exposed on the PMC NC surface, we would change possibly the energy level of π* orbital by using chemical modification so as to control and tune physicochemical properties of PMC NCs. Surface-modified PMC NCs have highly potential, and would open novel fields from the viewpoints of the surface science in complex material application.

## 2. Result and discussion

### Structural analysis of [{Cu_2_(μ-Br)_2_(PPh_3_)_2_}(μ-bpy)]_*n*_ NCs

**Fig. 2:**
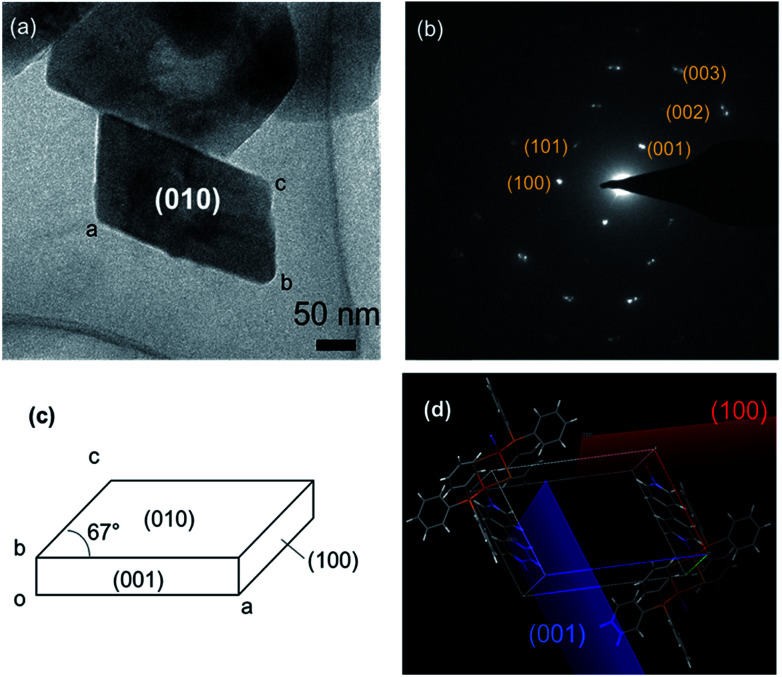
(a) TEM image of a single PMC NC viewed in the (010) direction, (b) electron beam diffraction patterns and the assignment of diffraction spots, (c) proposed crystal morphology and facet of PMC NC, and (d) the unit cell of PMC in a crystal state evaluated by X-ray single crystal structural analysis.

Fig. 2(a) shows the TEM image of polymer–metal complex [{Cu_2_(μ-Br)_2_(PPh_3_)_2_}(μ-bpy)]_*n*_ NCs (hereinafter, called “PMC NCs”) prepared by the heterogeneous reaction process.^13–15^ The present PMC NCs were obtained as a parallelogram-like plate with *ca.* 200 nm in size, and have high crystallinity from powder XRD patterns (Fig. S1†). The XRD pattern of PMC NCs corresponded with that of bulk crystal.

In order to characterize the crystallographic planes, that is to say, facet, of these PMC NCs, the selected-area electron beam diffraction patterns were obtained, and then the diffraction spots were assigned as shown in Fig. 2(b). The combined use of the simulation softs, “Crystal Maker X” and “Single Crystal 3”, has predicted the well-developed of (010) plane as a facet. The (010) plane projection drawing and simulation of electron beam diffraction patterns were indicated in Fig. S2.† In addition, it has become apparent from X-ray single crystal structural analysis^3^ that the polymer main chain of PMC extends along *b*-axis. These experimental facts mean that the terminal ends of polymer main chains would be exposed on the (010) plane as dangling bonds. As a result, Fig. 2(c) demonstrates the proposed crystal morphology and facet of PMC NCs. Furthermore, the unit cell as illustrated in Fig. 2(d) exhibits the surface of these (001) and (100) planes would be covered with bulky phenyl group of triphenylphosphine (PPh_3_). In other words, the (010) plane in the PMC NCs has highly potential to react and/or interact with a specified surface modifier.

However, one cannot still identify chemically terminal structure on specific facet, that is, (010) plane, in the present PMC NCs. So, as discussed in the next section, the identification of terminal structure was attempted to chemically visualize by the use of PB nanoparticles (NPs) as a probe.

### Surface modification and analysis of [{Cu_2_(μ-Br)_2_(PPh_3_)_2_}(μ-bpy)]_*n*_ NCs by using PB NPs

**Fig. 3:**
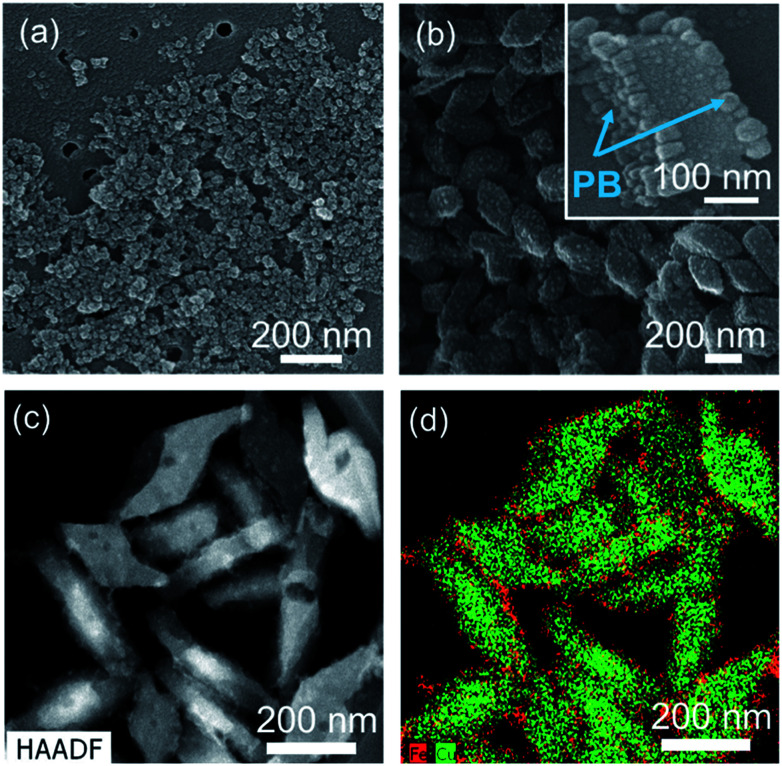
(a) SEM image of PB NPs, (b) SEM and (c) TEM images of PB-modified PMC NCs, and (d) the corresponding EDS mapping. The inset in (b) is the magnified image of (b). The average thickness of PMC NCs is about 40 nm, but we selected thick PMC NCs in the inset of (b) in order to view clearly the anisotropic adsorption of PB NPs. In the EDS mapping, red and green points indicate Fe atoms of PB and Cu atoms of PMC, respectively.

As above-mentioned in Fig. 2, the terminal ends would be located on the (010) plane of PMC NCs as dangling bonds, and there are the three possibilities for terminal ends, according to main chain structure of PMC NCs:^1^ 4,4′-bipyridine end-group at both terminal ends,^2^ unit of bromine-bridged Cu–PPh_3_ complex at both terminal ends, and^3^ 4,4′-bipyridine end-group and unit of bromine-bridged Cu–PPh_3_ complex at each terminal end. In order to reveal the terminal ends on the (010) planes, we have tried to modify the surface of PMC NCs using PB NPs as a probe. The size of PB NPs was about 10 nm from SEM observation as shown in Fig. 3(a). PB NPs contains Fe ions, and can chemically bond with a ligand and/or end-group having nitrogen terminal, for example, amine and pyridine groups.^16,17^ If 4,4′-bipyridine ligands are exposed on the (010) plane of PMC NCs, PB NPs would be selectively adsorbed on the PMC NCs. Even if bromine-bridged Cu–PPh_3_ unit is exposed on (010) surface of PMC NCs, we should note that the bond length of cyano group (–CN) on the surface of PB NPs is too short to coordinate on Cu ions in Cu–PPh_3_ unit, because the surrounding of Cu ion is quite bulky due to bromine-bridge and PPh_3_ units.

Actually, from DLS measurement, the average size of the resulting PB-modified PMC NCs was increased by *ca.* 100 nm after adding PB NPs. The PB-modified PMC NCs were also dispersed stably, because PB NPs would interact with PMC NCs with strong affinity, due to surface charge effect. In contrast, heterogenous aggregation was induced, when other metal NPs, for example Au and Ag, were added. That is, PB NPs were pretty suitable as a modifier for PMC NCs. In addition, the Zeta-potential of PMC NCs was changed from +30 mV to −30 mV before and after the addition of PB NPs. This result implies that the surface properties of PMC NCs have been considerably changed with the addition of PB NPs. Actually, the SEM image in Fig. 3(b) demonstrates that the PB-modified PMC NCs had rough surface, compared to as-prepared PMC NCs as shown in Fig. 1(b). One can confirm successfully the detailed surface structure of PB-modified PMC NCs in the magnified SEM image of the inset in Fig. 3(b). Interestingly and expectedly, PB NPs were selectively deposited only on the “specific two planes” of PMC NCs. Fig. 3(c) and (d) indicate TEM image and EDS mapping of PB-modified PMC NCs, which “stood” with (001) or (100) planes down. In other words, we could see (010) plane of PB-modified PMC NCs. The red dots, corresponding to Fe atoms in PB NPs, were characteristically located around the green dots area based upon Cu atoms in PMC NCs. Namely, not unit of bromine-bridged Cu–PPh_3_ complex but 4,4′-bipyridine ligand is considered reasonably to be exposed as terminal ends on the (010) plane in the present PMC NCs. Similarly, Y. Fujiki *et al.* reported l-cystine bulk crystal modified with Au NPs as a suitable probe.^1^ In this case Au NPs were selectively adsorbed, due to electrostatic interaction, on the specific surface area covered with the zwitterionic end-groups.

**Fig. 4:**
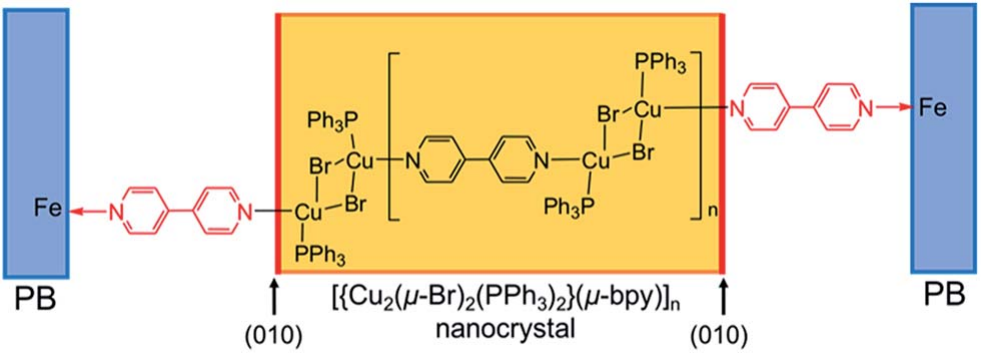
Schematically speculated terminal structure on specific facet in PB-modified PMC NCs.

Fig. 4 illustrates the proposed terminal structure on the specific facet of (010) plane in PB-modified PMC NCs.

## 3. Conclusions

We have successfully determined the crystallographic orientation of the present PMC NCs by means of TEM observation and analysis of electron beam diffraction pattern. It was found that the (010) plane has grown well, and that the terminal ends of main chains would be exposed and located on the (010) plane as a dangling bond. So, we have tried to modify chemically PMC NCs by using PB NPs, and then could “visualize” skillfully the specific surface and/or facets. Actually, PB NPs were selectively adsorbed on the (010) plane from SEM and TEM observation, and the corresponding EDS mapping. Consequently, it has become apparent that 4,4′-bipyridine ligands having nitrogen-terminal is exposed on the (010) plane of PMC NCs. In general, the analytical diffraction methods to evaluate crystal structure and/or unit cell have been established so far.^18,19^ In addition, STEM-EDS mapping^20^ provides the distribution profiles of concerned elements inside bulk crystals and NCs. However, it is so limited and difficult to assign and determine “terminal chemical structure” and/or “terminal chemical spices” exposed on the surface of bulk crystals and NCs. On the contrary, the present method, using suitable nanoparticles as a probe, is very simple, but can effectively and usefully visualize and characterize the specific surface structure rationally, even though one would assume or speculate surface chemical species in advance so as to reasonably select a probe. We now expect that PMC NCs would provide some possibility toward novel physicochemical properties induced by unique surface modification in the near future.

## 4. Experimental section

### Materials

Triphenylphosphine (PPh_3_), 4,4′-bipyridine, CuBr, Fe(NO_3_)_3_·10H_2_O, Na_4_Fe(CN)_6_·10H_2_O, and all solvents were commercially available. All chemicals were used as received.

### Fabrication of [{Cu_2_(μ-Br)_2_(PPh_3_)_2_}(μ-bpy)]_*n*_ NCs

[{Cu_2_(μ-Br)_2_(PPh_3_)_2_}(μ-bpy)]_*n*_ NCs were fabricated by the already-established heterogeneous reaction process.^13–15^ A 200 μL of acetone solution of PPh_3_ (0.5 mg, 2 μmol) was injected into vigorously stirred 10 mL of aqueous solution of 4.4′-bipyridine (0.6 mg, 4 μmol), so that PPh_3_ nanocrystals were formed and dispersed in a 4,4′-bipyridine aqueous solution. Subsequently, 200 μL of acetonitrile solution of CuBr (0.3 mg, 2 μmol) were added dropwise into the above dispersion liquid. The color of the dispersion liquid was changed from white to pale yellow. The obtained pale yellow NCs were filtered, washed with acetone, and re-dispersed in distilled water. [{Cu_2_(μ-Br)_2_(PPh_3_)_2_}(μ-bpy)]_*n*_ NCs were almost quantitatively obtained over 90% yields. Anal. calcd for C_46_H_38_Br_2_Cu_2_N_2_P_2_: C, 57.10; H, 3.96; N, 2.89. Found: C, 57.36; H, 4.03; N, 2.87.

### Synthesis of water-dispersible Prussian blue (PB) nanoparticles (NPs)

Water-dispersible PB NPs were prepared according to the following procedures as described elsewhere.^21^ A 3 mL of aqueous solution of Fe(NO_3_)_3_·10H_2_O (1.6 mg, 4.0 mmol) was added into an aqueous solution of 6 mL of Na_4_Fe(CN)_6_·10H_2_O (1.5 mg, 3.0 mmol), stirred for 5 min at room temperature, and then blue precipitates were formed. The blue precipitates were filtered off, washed with methanol, and dried *in vacuo*. Subsequently, the precipitates (0.4 g, 0.35 mmol) were suspended into an 8 mL of distilled water, and Na_4_Fe(CN)_6_·10H_2_O (0.06 g, 0.12 mmol) was further added into this suspension. The suspension was completely stirred at ambient temperature until changing to transparent deep-blue color.

### Fabrication of PB-modified [{Cu_2_(μ-Br)_2_(PPh_3_)_2_}(μ-bpy)]_*n*_ NCs

A 3 μL of PB NPs suspension liquid was added into 10 mL of [{Cu_2_(μ-Br)_2_(PPh_3_)_2_}(μ-bpy)]_*n*_ NCs dispersion liquid at ambient temperature, and then the mixture was allowed to stand for 3 hours.

### Measurements for characterization

Structural and morphological characterization for the present [{Cu_2_(μ-Br)_2_(PPh_3_)_2_}(μ-bpy)]_*n*_ NCs was performed by scanning electron microscope (SEM; JSM-6700F, JEOL) and transmission electron microscope (TEM, Titan 80-300, FEI, operated at 300 kV). The electron beam diffraction pattern was also obtained by TEM (Titan 80-300, FEI, operated at 300 kV). EDS analysis was conducted using TEM (Titan 3 G2 60-300, FEI, operated at 60 kV). Dynamic light scattering (DLS) and Zeta potential measurement were performed by Zetasizer Nano-ZS (Malvern). The unit cell of [{Cu_2_(μ-Br)_2_(PPh_3_)_2_}(μ-bpy)]_*n*_ in a crystal state was evaluated by using simulation soft “Crystal Maker X” and “Single Crystal 3” released by HULINKS Inc.

## Conflicts of interest

There are no conflicts to declare.

## Supplementary Material

RA-008-C8RA02165A-s001
