# Supplementary material for: Attempt to visualize terminal structure on a specific facet in polymer–metal complex nanocrystals
Source: RSC Adv. 2018 May 3;8(30):16406–9. doi: 10.1039/c8ra02165a (PMC9080293; doi:10.1039/c8ra02165a)
Supplement: RA-008-C8RA02165A-s001 [file RA-008-C8RA02165A-s001.pdf]

This journal is © The Royal Society of Chemistry 2018

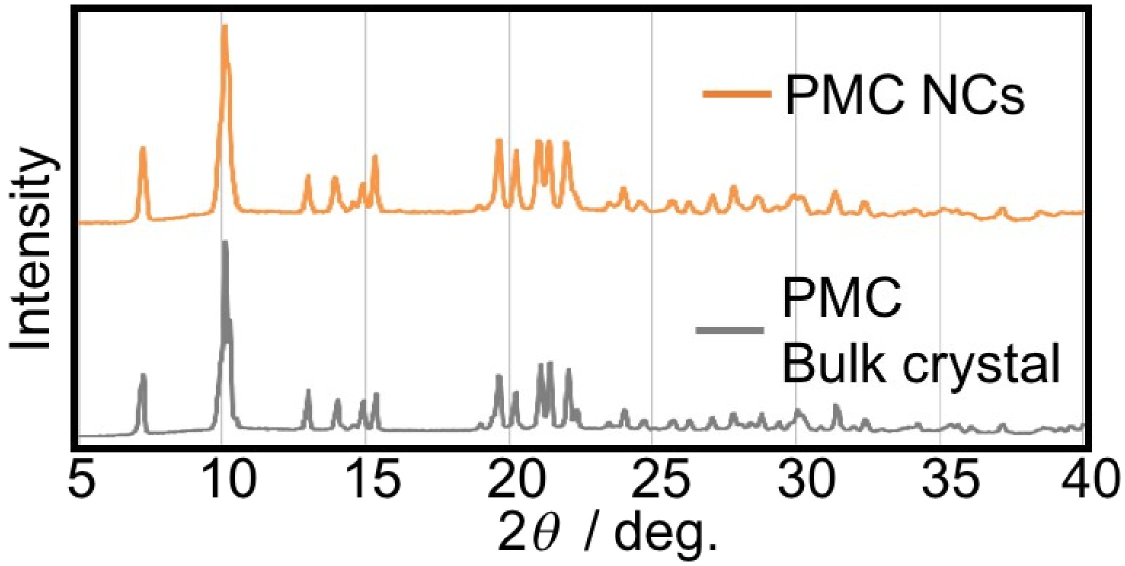

Fig. S1 Powder XRD patterns of $[\{Cu_2(\mu\text{-}Br)_2(PPh_3)_2\}(\mu\text{-}bpy)]_n$, called "PMC" in the Text, nanocrystals (NCs) and bulk crystal.

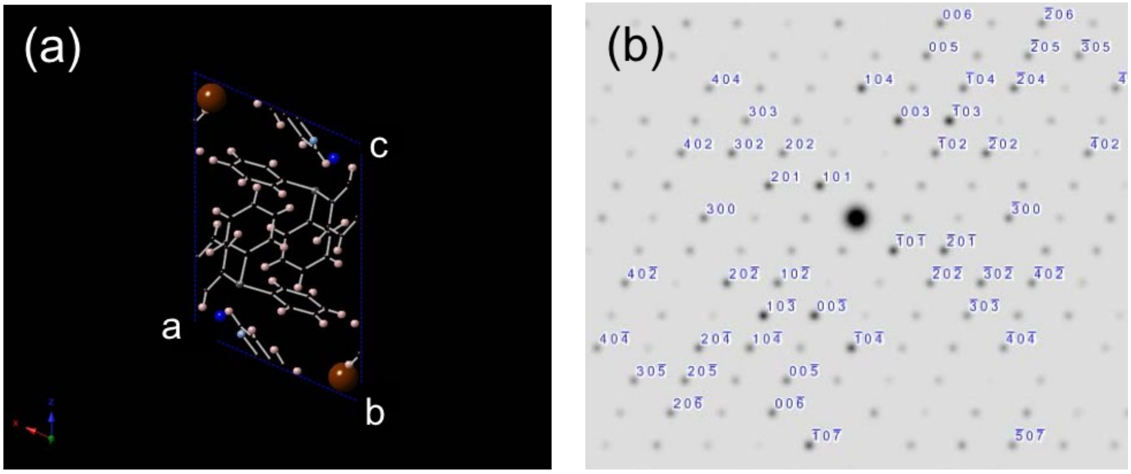

Fig. S2 (a) (010) plane projection drawing and (b) simulation of electron beam diffraction patterns in $[\{Cu_2(\mu\text{-}Br)_2(PPh_3)_2\}(\mu\text{-}bpy)]_{n,}$ obtained by the simulation softs, “Crystal Maker X” and “Single Crystal 3”.

.
